# Balance Functional Assessment in People with Visual Impairment

**DOI:** 10.1515/hukin-2015-0096

**Published:** 2015-01-12

**Authors:** Izabela Rutkowska, Grzegorz Bednarczuk, Bartosz Molik, Natalia Morgulec-Adamowicz, Jolanta Marszałek, Kalina Kaźmierska-Kowalewska, Krzysztof Koc

**Affiliations:** 1 Department of Sports for Individuals with Disabilities, Department of Theo Movement, Józef Piłsudski University of Physical Education, Warsaw, Poland; 2 Department of Adapted Physical Activity,,Józef Piłsudski University of Physical Education, Warsaw, Poland; 3 Department of Theory and Methodology in Teaching Movemen, Józef Piłsudski University of Physical Education, Warsaw, Poland; 4 Educational Center for Bind Children in Laski, Poland

**Keywords:** body balance, people with visual impairment, functional tests

## Abstract

The aims of this study were twofold: to assess the level of balance of people with visual impairment against the BOT-2 standard scores for the able-bodied, and to identify in which trials subjects had the greatest difficulties in maintaining balance with respect to the degree of vision loss and age categories. One hundred twenty-seven subjects with visual impairment aged 6–16 years, participated in the study (68 girls and 59 boys). The division for partially sighted people (61) and the blind (66) was made according to the WHO classification. Functional balance assessment was made using a balance subtest from the Bruininks-Oseretsky test. Significant relationships were noticed between age and the level of balance (χ2 = 8.35 p <0,05), as well as between the degree of vision loss and the level of balance (χ2 = 24.53 p <0,001). The level of balance of almost all blind subjects was below (20%) or well-below (60%) the average for the able-bodied. The subjects’ ability to maintain balance was not dependent on gender and was associated primarily with the degree of visual impairment and age. Partially sighted people had better balance than the blind and the decrease in visual acuity resulted in reduction of balance skills. The lowest level of balance was observed in blind students aged 7–11 years. Elaborating physical fitness improvement programs for children and adolescents with visual impairment, diversity of age, the degree of vision loss and limitations of ablility to maintain balance should be taken into account.

## Introduction

Visual stimuli ensure creation of a surrounding reference system. On the basis of this system, information about balance deviations is analysed and their corrections to prevent loss of body balance are possible. A well-functioning organ of vision complements activities of the vestibular system (Held-Ziółkowska, 2006), thus creating a postural control system. Visual impairment (VI) causes partial or total limitation of the use of the spatial reference system and precise correction of body alignment, thereby negatively affects the maintenance of body posture and balance (Wade and Jones, 1997).

The ability to maintain balance, in view of process complexity, can be examined at the physiological, as well as functional level (Browne and O’Hare, 2002). Physiological assessment comprises evaluation of various elements of the balance system, all reception parts, as well as integration and effector parts. Functional assessment allows testing real movement possibilities of subjects. The disadvantage of the functional assessment is that it is often quite subjective, and moreover, precise determination of which part of “the balance system” and to what extent is disordered, is impossible (Browne and O’Hare, 2002; Nowotny et al., 2009). Some authors claim that functional balance assessment of people with VI may be more reliable than using the stabilographic method, in which obtained results have minor significance for daily functioning of the blind and the possibility of elaboration of treatment programs. According to Sherrill (2004), for a child a movement task meets the functionality criteria if it is a typical daily activitiy including physical education and extra-curricular classes.

Balance assessment of people with VI has been the subject of numerous researches (Gawlik, 2008; Horvat et al., 2003; Poliszczuk and Rutkowska, 2006). The literature review revealed that the results of studies comparing the level of this ability of the blind to the partially sighted and to the able-bodied, as well as factors affecting the ability to maintain body balance of participants with VI, were equivocal.

Houwen et al. (2009b) in a comprehensive literature-review article showed that the evidence to conclude that the degree of vision loss correlated with the ability to maintain static balance was insufficient (Houwen et al., 2007; Houven et al., 2008; Juodžbalienė and Muckus, 2006), as in some studies the blind achieved results similar to, or better than the partially sighted, and sometimes better even than the able-bodied (Johnson-Kramer et al., 1992; Nakata and Yabe, 2001; Poliszczuk and Rutkowska, 2006). Although visual perception eases control of static balance, the information derived only from the vestibular system and proprioceptors enables acquiring multiple motor skills without the participation of vision, especially those in which locomotion and dynamic balance do not play a primary role. This means that blind children develop alternative strategies to compensate vision loss already at early stages of life.

The analysis conducted by Houwen et al. (2009b) showed weak evidence that the degree of VI affected the ability to maintain body balance in dynamic conditions. The greater the limitations of functions of the oculomotor apparatus and reduction of visual acuity and the angle (which means incorrect and/or insufficient delivery of visual stimuli), the worse the balance of subjects in dynamic conditions, as compensatory mechanisms were not able to replace the eyesight efficiently (Nakata and Yabe, 2000; Pereira, 1990; Wade and Jones, 1997). For example, mastering bipedal locomotion, which requires the ability to withstand the force of gravity and maintain balance in dynamic conditions, was far more difficult for blind children than static balance (Brambring, 2006). On the basis of research results of Adelson and Freiberg (1974), Levitt (2003) and Brambring (2007), it could be inferred that the development process of balance, particularly of blind children, may be delayed or proceed differently in comparison to able-bodied peers.

The necessity of diagnosing balance and detecting possible disorders result from, inter alia, the primary role of this motor skill in mastering independent locomotion and many other physical activities by children. Authors of this study used one of the subtests of the BOT-2 battery of motor development tests, which evaluates functional balance and can be conducted on able-bodied children and adolescents, as well as with certain types of disability. Detailed analysis of score profiles could help people, who are involved in improving physical fitness of children and adolescents with VI, with proper selection of exercises that shape balance. The literature has not revealed such analyses, as functional balance assessment has been rarely performed and balance evaluation using the stabilographic method dominated.

The purpose of this study was to determine the influence of selected factors (gender, age, degree VI) on body balance of people with VI, and moreover, to analyse the level of motor skills against standards of the BOT-2 for able-bodied people. An additional objective was to identify tasks in which subjects had the greatest difficulties in maintaining balance in relation to the degree of vision loss and age of the participants.

## Material and Methods

### Participants

The study was conducted on 127 students of four educational centres for blind and partially sighted children in Poland. The inclusion criteria comprised: minimal disability, which was consistent with the WHO classification (WHO 2012) i.e. visual acuity of less than 6/18, and subjects’ participation in physical education classes (2–3 times a week). The exclusion criteria such as nervous system disorders, structural changes within the musculoskeletal system and intellectual limitations, were determined on the basis of an interview and medical records. All subjects provided written consents from parents or legal guardians to participate in the study and had the right to insight into their medical records.

The division of the participants into the partially sighted (n=61) and the blind (n=66) was consistent with the WHO classification, described in ICD-10 (WHO 2012), i.e. the partially sighted had moderate or severe VI (category 1, 2) - visual acuity of less than 6/18 and not better than 3/60, and the blind (category 4 and 5) - visual acuity of less than 1/60. The measurements were performed in the better eye with best possible correction. Qualifications were based on data from the documentation of ophthalmologists and vision therapists from educational centres for blind and partially sighted children.

The average age of participants was 12.6 ± 2.7 (girls 12.6 ± 2.4 and boys 12.5 ± 2.6). Two age groups of subjects were distinguished in this study:

- Younger participants 6–11 years (mean 9.3±1.6 years), total number n=56 (blind n=31, partially sighted n=25)
- Older participants: 12–16 years (mean 14.1±1.4 years); total number n=71 (blind n=35, partially sighted n=36)

### Procedures

Functional balance assessment was made using the balance subtest from the Bruininks-Oseretsky Test of Motor Proficiency, Second Edition (BOT-2), hereafter referred to as the BOT-2 balance subtest (Bruininks and Bruininks, 2005). The test was administered at a specially prepared area consisting of a walking line, a balance beam (special beam) and a shield hanging on the wall (reference point for the partially sighted). Adaptations for the blind based on using velcro tape (hook-and-loop tape) sticked to the floor, so that it was palpable by feet. Moreover, in some cases in addition to verbal description of the test, nonverbal method was used, the so-called “brailing”. The researcher used his/her own body to demonstrate the starting position and the sequence of movements while a blind student followed the body motion with his/her fingers. The subjects performed 9 tasks:

1. Standing with feet apart on a line – eyes open.
2. Walking forward on a line.
3. Standing on one leg on a line – eyes open.
4. Standing with feet apart on a line – eyes closed.
5. Walking forward heel to toe on a line.
6. Standing on one leg on a line – eyes closed.
7. Standing on one leg on a balance beam – eyes open.
8. Standing heel to toe on a balance beam.
9. Standing on one leg on a balance beam – eyes closed.

All subjects were blindfolded during tasks with eyes closed (according to the standardization of the BOT-2) also the blind, as some of them had light perception. Every task was performed twice, but only if in the first trial the subject did not receive the maximum number of points.

The balance level was evaluated on the basis of the BOT-2 standards using the so-called descriptive category and age equivalent (Bruininks and Bruininks, 2005). Results of 9 tasks were evaluated on a scale from 1–4 points (the maximum result of the balance subtest was 36 points). According to the methodology described by the authors, the point scores of the 9 tasks were summed to produce a total point score. The total point score was compared with the gender and age frame of reference to achieve the so-called age-matched scale score, which was assessed using the descriptive category and age equivalent. The descriptive category (average range, below the average, well-below the average and above the average) expresses approximate distance from the 50th percentile that was calculated on the basis of screening tests for the able-bodied population. The age equivalent is used to estimate whether the stage of balance development is consistent with the average pace of the skill development for the population and to identify the differences.

Subjects’ body height and mass were measured using an electronic height and weight scale in accordance with generally accepted principles (Table 1). The accuracy of body height measurement was 0.5 cm and of body mass 0.1 kg.

The study was performed within statutory activity of the Józef Piłsudski University of Physical Education in Warsaw under the project title *Evaluation of the functional body balance of people with visual impairment*. A member of the research team invited individuals to participate in the research and provided them with appropriate verbal information about the study. The subjects had an opportunity to ask questions about the objectives and procedures of the study. Verbal and written consent was obtained before the group assignment began (in accordance with the Declaration of Helsinki); this also met the criteria for informed consent as outlined by the institutional Biomedical Research Ethics Committee.

### Statistical analysis

Normal distribution of analysed variables was examined using the Shapiro-Wilk test, and the assumption of equal variances was tested using the Levene’s test. The test-retest method, the Pearson’s correlation coefficient and the interclass correlation coefficient (ICC) were applied to assess reliability of the BOT-2 balance subtest.

The significance of differences in the BOT-2 balance subtest total point scores in both groups of participants was evaluated using three-way analysis of variance (age × gender × degree of VI, taking into account division into the partially sighted and the blind). The significance of differences between pairs of averages was tested using the Tukey’s test (post-hoc). The relationship between the two age groups, genders, the degree of VI and the level of balance descriptive category was estimated using the chi-square test.

In addition to the assessment of the diversity of the BOT-2 balance subtest total point scores, also difficulty of each task of the subtest was analysed. Due to the diversity in the maximum number of points for different tasks that subjects could obtain, the comparison was made after bringing the results to a common scale by dividing the observed value by the maximum point value possible to be achieved. Considering the discreet nature and small range of participants’ test results variability, the differences between the results obtained by the distinguished age groups and in the disability categories were examined using non-parametric methods i.e. Friedman’s analysis of variance and the Wilcoxon test. In all analyses, the statistical significance was set at the level of *α*=0.05. The results were processed using the Statistica 9.0 PL program for Windows (StatSoft, Inc., 2009).

## Results

Repeatability of the BOT-2 balance subtest results was evaluated using comparison of mean values obtained in two measurements conducted at an interval of two weeks (1st measurement −12.45 ± 7.22; CV = 0.58; 2nd measurement 13.20 ± 6.33; CV = 0.48). The distributions of scores in both measurements did not differ from normal distribution. On the basis of the Pearson correlation coefficient, the results revealed high repeatability (r = 0.915; p <0.0001; 95% CI lower 0.797– upper 0.963).

**Figure 1:**
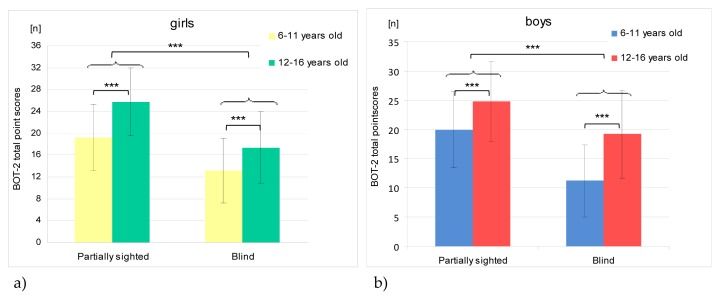
Mean values of total points scores in the BOT-2 balance subtest characterizing balance of girls (a); (n=68) and boys (b); (n=59) with division into age and level of vision loss categories. ***p < 0,001

The analysis of variance was used to examine the effect of 3 factors on the final score of the BOT-2 balance subtest, i.e. gender, age and the degree of visual impairment (the blind and the partially sighted). No significant interactions were noted (F1,127= 1.32; p> 0.5) between these factors taking into consideration the division into blind and partially sighted people. Gender had no significant influence on the total score of BOT-2 balance tasks (girls 18.9 ± 7.37 points, boys 20.1 ± 7.51 points). The total point score depended on the degree of vision loss and age of the participants. This means that, regardless of gender, the partially sighted (22.8 ± 6.61 points) achieved better results than the blind (16.8 ± 7.41 points). Participants aged 12–16 years (21.6 ± 6.87 points), regardless of gender and the degree of vision loss, obtained higher scores of the BOT-2 balance subtest than those aged 6–11 years (15.8 ± 7.23 points) (Figure 1a, b).

In this study the relationship between 3 factors (gender, age, and the degree of vision loss) and the level of the balance descriptive category was evaluated. The results confirmed the outcomes obtained for the uncategorised values. The relationship between the level of balance and gender was not significant (χ2 = 1.54 p> 0.05), however, it was significant between the level of balance and age (χ2 = 8.35 p <0.05), as well as between the level of balance and the degree of vision loss (χ2 = 24.53 p <0.001).

**Figure 2:**
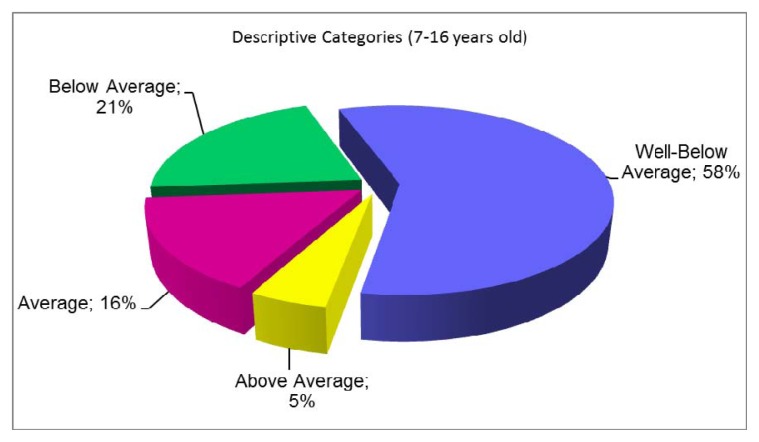
Percentage distribution of different descriptive categories describing the level of balance of subjects with IV (n=127) in relation to the standards for able-bodied people (Bruininks and Bruininks, 2005)

Analysis of the balance level demonstrated that more than 60% of participants, regardless of gender, achieved results well-below average, and more than 20% below average for the able-bodied population. Percentage distribution of various categories that describe the balance level against the BOT-2 standards for able-bodied people (Bruininks and Bruininks, 2005) is presented in Figure 2. Balance ability of about 95% of younger participants and 84% of older participants was below or well-below average for the able-bodied. However, slightly fewer older children (57%) than younger (76%) had balance assessed well-below average. Almost all the blind subjects were characterized by balance below (22%) or well-below average (75%) when compared to the able-bodied. Although almost half of the partially sighted obtained results corresponding to values well-below average, 25% of them reached a score at the level of average or above average.

**Figure 3:**
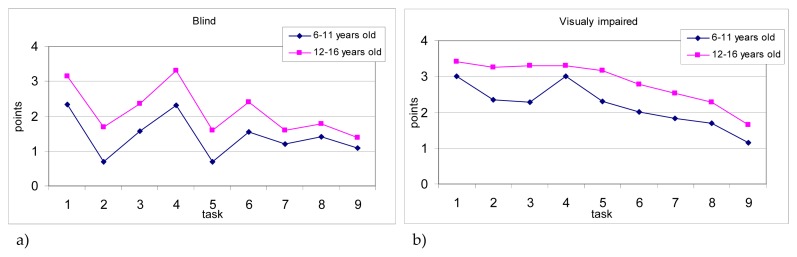
Mean scores, obtained in each task of the BOT-2 balance subtest by a) the blind: younger (n = 31) and older (n = 35) participants b) the partially sighted: younger (n = 25) and older (n = 36) participants. Tasks 1. Standing with feet apart on a line – eyes open. 2. Walking forward on a line. 3. Standing on one leg on a line – eyes open. 4. Standing with feet apart on a line – eyes closed. 5. Walking forward heel to toe on a line. 6. Standing on one leg on a line – eyes closed. 7. Standing on one leg on a balance beam – eyes open. 8. Standing heel to toe on a balance beam. 9. Standing on one leg on a balance beam – eyes closed

Analysis of score profiles of mean values of the balance subtest, in VI and age categories, revealed that results of tasks of younger partially sighted participants were significantly diverse (F8,25= 88.42; p <0.001). The highest assessed tasks were standing with feet apart on a line performed with eyes open (task 1) and eyes closed (task 4). Statistical analysis did not reveal differences between task 1 and task 4, whereas these two tasks demonstrated significantly higher scores in comparison to the other tasks. The lowest score was noted for task 9 (standing on one leg on a balance beam with eyes closed), and the score was significantly lower compared to the other tasks. Mean values in each task obtained by the partially sighted aged 12–16 years significantly differed (F8,36= 121.46; p <0.0001). On the basis of results analysis, it can be concluded that the degree of difficulty of tasks for this group of participants was consistent with the score profile reported by authors of the test, i.e. the difficulty increased in subsequent tasks (Figure 3b). For both, younger and older partially sighted participants, the most difficult task was the last one - standing on one leg on a balance beam with eyes closed.

Similarities were observed in the score profiles between the two groups of blind subjects, (younger and older), but mean values of these two groups, in each task, differed significantly (F8,31= 92.38; p <0.0001; F8,35= 101.36; p <0.0001, respectively). Results obtained by older participants in each task were slightly higher in comparison to the younger (Figure 3a). Regardless of age, the blind received the highest scores in task 1 and 4, i.e. standing with feet apart on a line performed with eyes open and closed, respectively. Moreover, results of these two tasks were significantly higher than in the other tasks. Younger blind participants received the lowest score for two walking on a line tasks (task 2 – free walking, task 5 - walking heal to toe), and furthermore, results of the two tasks were significantly lower in comparison to the other tasks. Younger blind subjects obtained low mean scores also in tasks performed on a balance beam (tasks 7, 8, 9) with results slightly over 1 point (max. 4 points). The blind aged 12–16 years, similarly to younger blind participants, gained the lowest score for both walking on a line tasks, and for all 3 tasks performed on a balance beam, with the results significantly lower compared to the other tasks, however, no differences were noticed between the weakest five tasks.

## Discussion

Due to the fact that studies concerning the assessment of reliability of the BOT-2 balance subtest among people with VI have not been found in the literature, the results’ repeatability of the test was evaluated in the pilot study. As recommended by Lieberman et al. (2006) and Houwen et al. (2009a), special adaptations were applied to the tasks, taking into consideration abilities of people with VI. One of important changes was to replace a painted line with a velcro tape (hook-and-loop tape) sticked to the floor, so that blind people could feel it under the soles of sport shoes. In addition, before starting a task, proper body alignment and movement were described in detail to all subjects, but the tasks were demonstrated using the so-called “brailing” to the blind and some partially sighted participants. The repeated tests revealed that the BOT-2 balance subtest was a reliable tool for assessing the ability to maintain balance of people with VI.

Review of the literature substantiated the need to verify such research issues as the identification of factors that may affect the ability to maintain balance of children and adolescents with VI and identification of motor tasks in which subjects had the greatest balance difficulties taking into account such factors as the degree of vision loss and age.

This study demonstrated that partially sighted people achieved better results than the blind, and the lower the visual acuity, the greater the difficulty with maintaining balance in functional tests. Evaluation of the results using the BOT-2 standards confirmed the above-mentioned outcomes and further expanded information revealing that 75% of blind and half of partially sighted participants had significantly worse balance than an average level of the skill of their able-bodied peers. This unequivocally confirmed that the VI negatively affected the ability to maintain balance factor. The results of this research are similar to the results presented in few publications, which indicated the relationship between the degree of VI (i.e. visual acuity and/or the visual field) and the ability to maintain balance. A lower level of the skill of the blind in comparison to partially sighted and able-bodied peers was corroborated particularly by the outcomes of studies that evaluated dynamic balance (Brambring, 2007; Ray and Wolf, 2010). In static conditions balance evaluation of people with VI largely depended on the position in which the test was conducted. The results obtained by people with VI in standing with feet together did not differ significantly from those achieved by the able-bodied (Horvat et al., 2003; Johnson-Kramer et al., 1992; Pereira, 1990). Along with a decrease of the base of support, balance skills of the blind and the partially sighted were more limited than those of the able-bodied (Wyver and Livesey, 2003; Giagazoglou et al., 2009).

Pereira (1990), on the basis of comparison of balance between people with mild, moderate and severe VI and the blind at the age of 6–13 years, stated that the highest level of ability to maintain balance had participants with visual acuity better than 0.1, with the visual field close to normal and without nystagmus. Ray and Wolf (2010) presented similar results and they noticed that the greater the degree of VI, the worse the ability to maintain balance in dynamic conditions.

The age of participants was also an important factor and its relationship with the ability to maintain balance in functional tests was evaluated in this study. Only few reports had concerned the evaluation of balance development in ontogenesis of people with VI (Adelson and Freiberg, 1974; Brambring, 2007). According to Brambring (2007), correct balance reactions of blind children in the early stages of life (0–6 years) appeared later in comparison to the able-bodied. Tasks such as standing on one foot, walking along a line, bending down to pick up an object, hopping on the spot on both legs, walking on tiptoes, that demanded the ability to maintain balance in dynamic conditions, revealed large and extreme differences in acquisition ages of approximately 10–20 months compared to able-bodied peers. Research on the population of people without disabilities concerning development of balance has proven that the 12-year-old children fully mastered the ability to control the standing position (Golema, 2002). According to Ljach (2003), the ability to maintain static and dynamic balance, which improves with age, reaches its maximum level among girls at the age of 17 years and boys at the age of 15 years.

This study demonstrated that participants aged 12–16 years, regardless of gender and the degree of vision loss, presented better ability to maintain balance in functional tests than those aged 6–11 years. Confirmation of this outcome are results obtained by Gipsman (1981) who compared the balance in static and dynamic conditions of blind and able-bodied people in two age groups: 8–10 years and 12–14 years. Regardless of age, children without disabilities achieved better results, whereas older blind children were characterized by better balance skills than younger blind children, which could mean that for the blind the use of proprioceptive information became more efficient with age. According to Gawlik (2008), in a group of blind subjects aged 10–18 years, the greatest balance improvement was observed among 16–18 year-old girls and 13–16 year-old boys. However, regardless of age, no significant differences were noticed between the first and repeated after 2 years measurements. It seems that longitudinal studies of balance development among children and adolescents with VI could be substantial.

Results of this research confirmed and extended the conclusion reached by Houwen et al. (2009b) based on a review of numerous studies. The authors showed weak evidence for lack of the relationship between the level of static balance and gender. In this study, it was found that gender did not affect the ability to maintain balance of participants with VI in functional tests i.e. in both static and dynamic conditions.

In this research also the level of difficulty of each of the BOT-2 balance subtest tasks was evaluated according to the degree of vision loss and age of participants, in order to identify in which tasks subjects had the greatest difficulty in maintaining balance. Detailed analysis of score profiles could help people, who are involved in improving physical fitness of children and adolescents with VI, with proper selection of exercises that develop balance. In the literature, no such analyses had been conducted, as for balance evaluation functional tests were rarely used, and the evaluation was mostly based on the stabilographic method in the basic position, standing on one leg, standing heel to toe or in dynamic conditions using artificially destabilizing stimuli caused by platform movements. It seems that the assessment of balance in functional tests is much more similar to daily activities of people with VI, and may be more significant for the practice of physiotherapists and physical education teachers than stabilographic research evaluation. In future studies, a relationship between balance results obtained in functional tests and in dynamic conditions using the stabilographic method should be examined.

This study revealed that, in accordance to age and the degree of vision loss (division into the blind and the partially sighted) the tasks difficulty profile created using obtained results varied. The younger blind participants had the greatest difficulty with maintaining balance in walking forward on a line in both, free and heel to toe walking. For the older blind subjects, the hardest tasks were in conditions of substantial reduction of the base of support, i.e. on a balance beam, and similarly to the younger group also in walking on a line. It seems that the observed deficiencies in the ability to maintain balance in some tasks may have a significant impact on locomotion efficiency of the blind, that was described by some authors (Skaggs and Hopper, 1996; Brambring, 2007).

The profiles of tasks results obtained by the partially sighted were diversified. Children aged 6–11 years had the greatest difficulty in maintaining balance during tasks in which at the same time reduction of both, the base of support (standing on one leg on a line and standing on a balance beam) and visual perception (blindfold), were applied. The tasks difficulty profile of older participants was consistent with the profile of able-bodied people, i.e. the difficulty increased in subsequent tasks (Bruininks and Bruininks, 2005). This may indicate that partially sighted people aged 12–16 years used reduced or residual visual stimuli to maintain body balance, and even though their balance skills were generally lower than the average level of able-bodied peers, the development of compensatory mechanisms (compared to the younger group) resulted in that the tasks difficulty profile of the BOT-2 balance subtest in this group was similar to the profile of people without disabilities.

Limitations of this study concerned the impossibility of more precise evaluation of the influence of the VI degree (visual acuity) on the ability to maintain balance, as on the basis of collected information, participants could be divided into two groups only: the blind and the partially sighted. It would be interesting to take into account the WHO classification of VI (6 categories), in which the main criterion of dysfunction is the level of visual acuity. However, to accomplish this, a larger number of subjects with diverse degrees of vision loss would be necessary. It seems important to examine in more detail the changes in balance, especially among the younger group (6–12 years) in order to verify whether it is a sensitive period in the development of this motor skill of children with VI. This would be significant for practical measures of physiotherapists and those involved in improving physical fitness of the blind and the partially sighted. On this basis, the question whether there are any differences in the development of balance in the ontogenesis between the able-bodied and people with VI could be answered.

## Conclusions

1. Visual impairment was a factor negatively affecting the ability to maintain balance in functional tests (BOT-2) - the participants’ balance level was below or well-below the average values determined for the population of able-bodied peers.
2. The ability to maintain balance of subjects in the BOT-2 functional tests was not associated with gender, but was related primarily to the degree of VI and age. Partially sighted people had a higher level of balance than the blind, and decreased visual acuity resulted in reduction of ability to maintain balance. In therapeutic activities, in terms of improving balance skills, particularly subjects with the greatest deficiencies, i.e. blind children (category 4 and 5 according to WHO Classification) at the age of 6–11 years should be included.
3. The balance subtest from the The Bruininks-Oseretsky Test of Motor Proficiency, Second Edition is a reliable tool for assessing the ability to maintain balance of people with visual impairment, with the use of adaptations described in the text.
4. A difficulty degree of the BOT-2 balance subtest tasks varied owing to the degree of vision loss (the blind and the partially sighted) and age. A detailed analysis of the results obtained in each task could help people who are involved in improving physical fitness of children and adolescents with VI, with deficiencies identification and proper selection of exercises that improve balance in both, static and dynamic conditions.

## Figures and Tables

**Table 1:** Mean values, standard deviation and range of values of basic anthropometric variables of people with VI in age and level of vision loss categories

Varablies	7–11 years (n = 56)		12–16 years (n = 71)		The blind (n = 66)		The partially sighted (n = 61)	

	Mean ± SD	Range	Mean ± SD	Range	Mean ± SD	Range	Mean ± SD	Range
Age [years]	9.3±1.6	6 – 11	14.1±2.5	12 – 16	11.6±4.6	6 – 16	11.9±4.8	6 – 16
Body mass [kg]	34.9±10.1	21.1 – 67.5	48.1±15.1	22.6 – 89.5	51.8±13.4	21.1 – 81.1	54.6±12.5	32 – 89.5
Body height [cm]	138±10.7	118 – 166	154.1±15.2	120 – 186	156.7±14.7	118 – 184	162.4±9.61	134 – 186
BMI	17.3±3.7	12.8 – 28.4	19.2±4.1	13 – 33.2	21.2±2.9	13.3 – 32.8	20.8±4.36	12.8 – 33.2
